# Perceived benefits and barriers towards exercise among healthcare providers in Ain Shams University Hospitals, Egypt

**DOI:** 10.1186/s42506-020-00042-1

**Published:** 2020-08-14

**Authors:** Hebat-Allah M. S. Gabal, Maha M. Wahdan, Waleed Salah Eldin

**Affiliations:** Department of Community, Environmental and Occupational Medicine, Faculty of Medicine, Ain Shams University, Cairo, Egypt

**Keywords:** Exercise, Prevalence, Benefits, Barriers, Healthcare providers

## Abstract

**Background:**

Physical inactivity is considered a problem with great impact on morbidity and mortality. There is a necessity to combat this behavior through an assessment of barriers and benefits perceived by subjects.

**Objectives:**

The current study aimed to measure exercise intention, in addition to identifying physical exercise predictors, including perceived barriers and benefits among healthcare providers (HCPs) in Ain Shams University Hospitals.

**Methods:**

A cross-sectional study was performed on a sample (*n* = 327) of healthcare providers (physicians and nurses) in Ain Shams University Hospitals in Cairo, Egypt, using anonymous self-administrated questionnaires and relevant scales.

**Results:**

Out of 327 healthcare providers approached, 52.6% of them were males and the mean age of participants was (29.34 ± 7.84 years). Physical exercise was reported by 44.6% of participants. The most significant factors associated with physical exercise engagement were male gender, participants with lower body mass index (BMI), and those with higher perceived “life enhancement subscale” benefit score and lower perceived exercise milieu and physical exertion barriers subscale scores. Exercisers showed significantly higher total overall (benefits and barriers) score compared to non-exercisers. Physicians showed a significantly higher total score compared to nurses.

**Conclusion and recommendations:**

The prevalence of regular exercise is low and inversely related to the female gender, BMI, Exercise milieu, and Physical exertion barriers subscale scores, and directly related to life enhancement benefit subscale score. Health education program about the benefits and barriers of exercise is recommended to encourage healthcare personnel to follow a healthy lifestyle as a role model to their patients and to act as health promoters to them.

## Introduction

Regular physical activity (PA) is considered a leading health indicator in Healthy People 2010 [1]. Regular participation in physical activity, especially in the form of exercise, leads to physical (physiological, morphological) and psychological benefits [2].

Lack of physical activity is known to be one of the most important risk factors leading to morbidity and mortality around the world leading to an estimated 3.2 million deaths globally [3]. Raised BMI could be one of the main risk factors associated with non-communicable diseases such as cardiovascular diseases, which were the leading reason for death in 2012; musculoskeletal disorders, and several types of cancer (including endometrial, breast, ovarian, prostate, liver, gallbladder, kidney, and colon). In 2016, over 1.9 billion adults were overweight; of these, over 650 million were obese [4]. WHO estimated that between 2 and 7% of healthcare spending in developed economies can be attributed to obesity [5].

Therefore, the World Health Organization (WHO) recommends that all adults ought to engage in regular exercise that is outlined as “any planned physical activity (e.g., brisk walking, aerobics, jogging, bicycling, swimming, rowing) performed to increase physical fitness. Such activity ought to be performed 3 to 5 times per week for 20–60 minutes per session” [6].

Although the awareness about these facts is prevalent among the community, it does not lead to greater participation in exercise, as it is a complex behavior, dependent on many factors, as personality (cognitive, emotional, motivational domain) and others in the environment, both physical and social [2].

Although the explanation of the reasons that hinder individuals from participating in PA is complicated and multi-factorial, it is considered a high priority to convince people to invest time and money in an activity. Participation in physical exercise depends on perceived benefits and barriers. Perceived benefits represent a supportive or reinforcing consequence of a behavior. They may be intrinsic (such as improved alertness or diminished fatigue) or extrinsic (such as social acceptance or financial awards). The motivational value of perceived benefits is based on outcomes of previous personal experiences or outcomes observed by others. Perceived barriers to action are associated with the obstacles encountered with undertaking a specific behavior as unavailability, inconvenience, expense, difficulty, time, or personal cost. Perceived barriers may either prevent the starting or initiation of a new activity or decrease commitment and adherence to current activity. Amelioration of different barriers and increasing the perception of benefits by individuals will increase participation [7].

Health care workers are responsible for counseling appropriate health behaviors including physical activity. Personal physical activity among physicians and nurses and its reflection on their body built influence to some degree their exercise counseling as they are considered a role model to their patients and community [8]. Therefore, healthcare workers may be seen as more truthful by their patients if they are following their own health promotion advice [9, 10].

To our knowledge, there is a scarcity in studies done among Egyptian healthcare workers addressing physical activity and factors associated with it. This study was conducted aiming to measure exercise prevalence, perceived barriers and benefits towards exercise among healthcare providers (HCPs) in Ain Shams University Hospitals to promote effectively for physical activity among healthcare providers and their patients.

## Material and methods

### Study design and setting

A cross-sectional study was carried out among healthcare providers (physicians and nurses) in Ain Shams University Hospitals in Cairo, Egypt.

### Study duration

The study was conducted from January 2018 to August 2019

### Sampling method

A convenience sampling technique was used.

### Sample size

It was calculated using PASS II program for sample size calculation and according to Jamil et al. (2015), the expected prevalence of moderate physical exercise among healthcare providers = 68%, assuming prevalence in study population = 70% ± 10%; sample size of 323 HCWs can detect this prevalence with 95% confidence level [11]. Taking into consideration a 10% drop out rate, the sample was exceeded to 355 participants. A response rate of 92% was achieved, so 327 healthcare workers were included in the study.

### Data collection tool

An anonymous, Arabic, self-administered questionnaire was used, delivered in written form to our study participants; the questionnaire was composed of the following:

1. *Socio*-*demographic data* (e.g., age, occupation, education). 
2. *Self*-*reported weight and height* (BMI was calculated as weight in kilograms divided by the square of the height in meters (kg/m^2^) and was classified according to WHO classification: underweight (<18.5 kg/m^2^), normal weight (18.5–24.9 kg/m^2^), overweight (25–29.9 kg/m^2^), and obese (≥30 kg/m^2^) [4]).
3. *Intention to physical activity*: Whether the participant exercised or intended to exercise regularly was assessed using the *Stages of Change* (*SoC*) *short*-*form questionnaire*. Regular exercise is defined according to WHO as “Regular exercise is any planned physical activity (e.g., brisk walking, aerobics, jogging, bicycling, swimming, rowing, etc.) performed to increase physical fitness. Such activity should be performed 3 to 5 times per week for 20–60 minutes per session. Exercise does not have to be painful to be effective but should be done at a level that increases your breathing rate and causes you to break a sweat.” Answer choices were categorized as not intending to exercise regularly (pre-contemplation stage), intending to exercise regularly in the next 6 months or 30 days (contemplation and preparation stage, respectively), and patients exercising regularly for less or more than 6 months (action and maintenance stage, respectively) [12, 13].

(4) *The Exercise Benefits and Barriers Scale* (*Adult Arabic Version*): (EBBS; Sechrist et al. 1987) consists of 43 items, with 14 items related to barriers and 29 items related to benefits, demonstrating the benefits and barriers that people associate with exercising. This scale is a 4-point Likert scale, from 1 (strongly disagree) to 4 (strongly agree). Barrier scale items are reverse-scored. The possible scores on the benefits scale ranged from 29 to 116 points, with higher scores indicating greater benefits. The possible range of scores on the barriers scale was 14 to 56 points, with a higher score indicating fewer perceived barriers. The total overall score was calculated by the addition of benefits and barriers scores. It ranged from 43 to 172 points. The higher the score, the more positive physical activity benefits were perceived in relation to physical activity barriers. Barriers include five categories: exercise milieu, time expenditure, physical exertion, family encouragement, and facility obstacles. Benefits fall under five categories as well: life enhancement, physical performance, psychological outlook, social interaction, and preventive health. The reliability of this scale was established and Cronbach’s alpha was 0.94, demonstrating good reliability; then, validation was done by other studies [14–20].

### Data management and analysis

Data were revised, coded, entered on a computer, and analyzed using SPSS package version number 20. Quantitative data were tested for normality and were normally distributed. Quantitative data were described as mean, standard deviation (SD), and range values. An independent *t* test was used for comparing quantitative variables between groups. Qualitative data were expressed as frequencies (*n*) and percentage (%). Chi-square and Fisher exact tests were used to test the association between qualitative variables. *p* value ≤ 0.05 was considered significant. Multivariate logistic regression analysis was performed for finding the predictors of exercise practicing. Significant variables in univariate analysis were included in the multivariate analysis model.

## Results

The mean age of the studied 327 healthcare providers was 29.34 ± 7.84 years and 52.6% of them were males and near half were overweight and obese (48.7%). About 44.6% of participants reported being currently regularly engaged in physical exercise. As regards exercise intention, 28.8% of participants were non-exercisers, but they had the intention to start; while 26.6% were non-exercisers and they did not have the intention to start (Table 1).

**Table 1:** Characteristics of healthcare providers (*n* = 327), Ain Shams University hospitals, Cairo, Egypt

	Number	Percent
**Age** (**years**) mean (SD) (min–max)	29.34 (7.84) (19–58)	
**Sex**		
**Male**	172	52.6
**Female**	155	47.4
**Occupation**		
**Nurse**	77	23.5
**Physicians**	250	76.5
**Marital status**		
**Single**	182	55.7
**Married**	128	39.1
**Divorced**	6	1.8
**Widow**	11	3.4
**Smoking**		
**No**	252	77.1
**Yes**	75	22.9
**Type of smoke**		
**Cigarette**	41	52.6
**Shisha**	33	42.3
**Others**	4	5.1
**Duration of smoking** (**years**) mean (SD) (min–max)	5.79 (3.81) (1–20)	
**BMI** (**kg/m**^**2**^) mean (SD) (min–max)	25.61 (4.24)	(16.38–42.72)
**BMI**		
**Underweight**	6	1.8
**Normal**	162	49.5
**Overweight**	114	34.9
**Obese**	45	13.8
**Exercise**		
**Yes**	146	44.6
**No**	181	55.4
**Exercise intention**		
**Exercise regularly > 6 months**	77	23.5
**Exercise regularly < 6 months**	69	21.1
**No but intend in next 30 days**	46	14.1
**No but intend in next 6 months**	48	14.7
**No and not intend in next 6 months**	44	13.5
**Do not know**	43	13.1

Regarding the association between respondents’ characteristics and physical exercise, male respondents were significantly more engaged in physical exercise compared with females. Also, there was a statistically significant difference between exercisers and non-exercisers as regard BMI with lower BMI among exercisers; this was also shown by the higher percentage of obese and overweight among non-exercisers. However, other factors such as occupation, marital status, and smoking showed no statistically significant relation with physical exercise engagement (Table 2).

**Table 2:** Association between participants’ characteristics and physical exercise

	Exercise				Test	*p*
	Yes	(%)	No	(%)		
**Age** mean (SD)	29.70 (8.20)		29.04 (7.54)		− .750^†^	.454
**Sex**						
**Male**	89	51.7	83	48.3	7.393^!^	.007*
**Female**	57	36.8	98	63.2		
**Occupation**						
**Nurse**	34	44.2	43	55.8	.010^!^	.921
**Physicians**	112	44.8	138	55.2		
**Marital status**						
**Single**	82	45.1	100	54.9	.327^#^	.955
**Married**	57	44.5	71	55.5		
**Divorced**	2	33.3	4	66.7		
**Widow**	5	45.5	6	54.5		
**Smoking**						
**No**	114	45.2	138	54.8	.155^!^	.694
**Yes**	32	42.7	43	57.3		
Type of smoke**						
**Cigarette**	17	41.5	24	58.5	.189^#^	.910
**Shisha**	15	45.5	18	54.5		
**Others**	2	50.0	2	50.0		
**Duration of smoking** mean (SD)	5.78 (3.28)		5.72 (4.22)		− .067^†^	.947
**BMI** mean (SD)	24.80 (3.99)		26.26 (4.34)		3.132^†^	.002*
**BMI**						
**Under weight**	5	83.3	1	16.7	12.177^#^	.007*
**Normal**	84	51.9	78	48.1		
**Over weight**	42	36.8	72	63.2		
**Obese**	15	33.3	30	66.7		

^†^Independent *t* test

^!^Chi-square test

^#^Fisher exact test

*Significant *p* value < 0.05

**more than one answer is allowed

Generally, as regards exercise benefit items as perceived by HCPs, the items with highest scores as reported by HCPs were decrease stress (mean = 2.94, SD 0.92), improve muscle strength (mean = 2.94, SD 1.9), improve body shape (mean = 2.92, SD 0.87), and mental health (mean = 2.91, SD 0.91) (Table 3).

**Table 3:** Exercise benefits statements and subscales scores in terms of exercise

	Total, mean (SD)	Exercise		Test^**†**^	*p*
		Yes, mean (SD)	No, mean (SD)		
**Enjoy exercise**	2.87 (0.95)	3.08 (0.96)	2.7 (0.91)	3.680	< .001*
**Decrease stress**	2.94 (0.92)	3.05 (0.94)	2.84 (0.9)	2.105	.036*
**Improve mental health**	2.91 (0.91)	3.05 (0.91)	2.8 (0.91)	2.571	.011*
**Personal accomplishment**	2.83 (0.8)	2.92 (0.81)	2.75 (0.78)	1.957	.051
**Feel relax**	2.75 (0.83)	2.87 (0.82)	2.65 (0.82)	2.383	.018*
**Improve wellbeing**	2.74 (0.83)	2.76 (0.83)	2.73 (0.84)	.334	.739
**Psychological outlook subscale score**	2.84 (0.7)	2.96 (0.72)	2.74 (0.66)	2.780	.006*
**Prevent heart attack**	2.84 (0.84)	2.95 (0.83)	2.75 (0.84)	2.208	.028*
**No high blood pressure**	2.75 (0.89)	2.79 (0.85)	2.71 (0.92)	.824	.411
**Live longer**	2.76 (0.85)	2.89 (0.83)	2.66 (0.85)	2.495	.013*
**Preventative health subscale score**	2.78 (0.63)	2.88 (0.61)	2.71 (0.64)	2.494	.013*
**Increase muscle strength**	2.94 (1.9)	2.95 (0.94)	2.93 (2.41)	.113	.910
**Increase physical fitness**	2.87 (0.91)	2.92 (0.97)	2.83 (0.86)	.893	.373
**Improve muscle tone**	2.84 (0.82)	2.94 (0.81)	2.76 (0.82)	1.943	.053
**Improve cardiovascular system**	2.90 (0.86)	2.96 (0.8)	2.85 (0.9)	1.190	.235
**Increase stigma**	2.77 (0.87)	2.86 (0.9)	2.7 (0.84)	1.597	.111
**Improve flexibility**	2.81 (0.85)	2.91 (0.89)	2.73 (0.82)	1.918	.056
**Physical endurance**	2.84 (0.8)	2.95 (0.79)	2.76 (0.79)	2.072	.039*
**Improve body shape**	2.92 (0.87)	3.01 (0.92)	2.85 (0.82)	1.748	.081
**Physical performance subscale score**	2.86 (0.7)	2.94 (0.67)	2.8 (0.71)	1.805	.072
**Contact people**	2.65 (0.82)	2.71 (0.85)	2.59 (0.8)	1.325	.186
**Meet new people**	2.67 (0.82)	2.69 (0.78)	2.65 (0.85)	.500	.618
**Good entertainment**	2.73 (0.79)	2.86 (0.81)	2.63 (0.76)	2.592	.010*
**Increase acceptance**	2.65 (0.77)	2.68 (0.8)	2.63 (0.75)	.642	.522
**Social interaction subscale score**	2.67 (0.59)	2.74 (0.61)	2.62 (0.57)	1.711	.088
**Disposition improved**	2.66 (0.8)	2.79 (0.7)	2.55 (0.85)	2.818	.005*
**Sleep well**	2.71 (0.9)	2.82 (0.89)	2.63 (0.9)	1.861	.064
**Decrease fatigue**	2.65 (0.82)	2.73 (0.81)	2.57 (0.83)	1.734	.084
**Self**-**concept**	2.76 (0.85)	2.88 (0.84)	2.66 (0.86)	2.263	.024*
**Mental alert**	2.76 (0.82)	2.82 (0.79)	2.71 (0.85)	1.198	.232
**Less tired**	2.76 (0.86)	2.88 (0.88)	2.65 (0.84)	2.433	.016*
**Improve work quality**	2.74 (0.81)	2.87 (0.81)	2.64 (0.8)	2.588	.010*
**Improve body function**	2.78 (0.83)	2.88 (0.84)	2.71 (0.82)	1.839	.067
**Life enhancement subscale score**	2.73 (0.61)	2.83 (0.61)	2.64 (0.6)	2.908	.004*

^†^Independent *t* test

*Significant *p* value < 0.05

On studying the relation between practicing exercise and perceived Exercise benefits subscales scores, we found a significant difference between exercising and non-exercising participants as regards psychological outlook benefit subscale scores, preventive health benefit subscale scores, and life enhancement benefit subscale score with exercising participants showing higher scores than non-exercising. However, no significant difference between exercising and non-exercising participants was detected as regards Physical performance subscale score and social interaction subscale score (Table 3).

While as regards exercise barrier items as perceived by the HCPs, the items with lowest scores (considered the highest barriers, as the score is reversed) as reported by HCPs were funny exercising clothes (mean = 2.58, SD 0.9), embarrassed to exercise (mean = 2.56, SD 0.89), and exercise takes time from family (mean = 2.52, SD 0.82) (Table 4).

**Table 4:** Exercise barriers statements and subscales scores in terms of exercise

	Total, mean (SD)	Exercise		Test^#^	*p*
		Yes, mean (SD)	No, mean (SD)		
**Too much time**	2.41 (0.83)	2.49 (0.83)	2.35 (0.83)	1.492	.137
**Time from family**	2.52 (0.82)	2.58 (0.8)	2.46 (0.84)	1.289	.198
**Take time**	2.45 (0.83)	2.54 (0.83)	2.37 (0.82)	1.866	.063
**Time expenditure subscale score**	2.46 (0.63)	2.54 (0.63)	2.39 (0.62)	2.040	.042*
**Far exercise place**	2.47 (0.87)	2.64 (0.88)	2.34 (0.84)	3.224	.001*
**Inconvenient facility schedules**	2.37 (0.73)	2.39 (0.72)	2.35 (0.75)	.518	.605
**Few exercise places**	2.43 (0.83)	2.47 (0.82)	2.4 (0.85)	.673	.501
**Embarrassed to exercise**	2.56 (0.89)	2.64 (0.97)	2.5 (0.81)	1.483	.139
**Costs to exercise**	2.43 (0.84)	2.63 (0.87)	2.28 (0.79)	3.847	< .001*
**Funny clothes**	2.58 (0.9)	2.68 (0.85)	2.5 (0.92)	1.821	.070
**Exercise milieu subscale score**	2.47 (0.50)	2.58 (0.54)	2.31 (0.59)	3.306	.001*
**Exercise is tiring**	2.36 (0.76)	2.47 (0.75)	2.27 (0.76)	2.317	.021*
**Exercise is fatiguing**	2.36 (0.77)	2.49 (0.74)	2.26 (0.78)	2.684	.008*
**Exercise is hard work**	2.50 (0.79)	2.63 (0.77)	2.4 (0.79)	2.602	.010*
**Physical exertion subscale score**	2.41 (0.58)	2.53 (0.54)	2.31 (0.59)	3.413	.001*
**No spouse encouragement**	2.47 (0.78)	2.49 (0.77)	2.45 (0.78)	.463	.644
**No family encouragement**	2.50 (0.83)	2.51 (0.83)	2.49 (0.83)	.297	.767
**Family discouragement subscale score**	2.48 (0.63)	2.50 (0.65)	2.47 (0.62)	.481	.631
**Total benefits and barriers overall score**	115.23 (18.17)	119.15 (19.15)	112.06 (16.73)	3.569	< .001*

^#^Independent *t* test

*Significant *p* value < 0.05

Regarding perceived barriers items and subscales scores in relation to practicing exercise, exercising and non-exercising participants were significantly different regarding all barrier subscales, except for the family discouragement subscale. Exercising HCPs showed lower barrier perception of physical exertion subscale, time expenditure subscale, and Exercise milieu subscale (Table 4).

Multivariate logistic regression was performed to predict exercise behavior. After including all significant variables (*p* < 0.1) in univariate analysis, it was found that gender, BMI, life enhancement benefit subscale score, exercise milieu subscale, and physical exertion barriers subscale score were the independent factors affecting practicing of exercise among studied group. Males were more liable to practice exercise compared to females (adjusted odds ratio = 2.06, CI = 1.28–3.33), participants with higher BMI were less likely to practice exercise (adjusted odds ratio = 0.92, CI = 0.869–0.975). Also participants with higher perceived benefit “Life enhancement subscale” score (adjusted odds ratio = 1.67, CI = 1.11–2.51), lower perceived “Exercise milieu subscale” barrier score (adjusted odds ratio = 1.8, CI = 1.12–3.1), and “Physical exertion subscale” barrier score (adjusted odds ratio = 1.58, CI = 1.04–2.46), were more likely to practice exercise (Table 5).

**Table 5:** Independent factors affecting exercise status by multivariate logistic analysis

	Adjusted odds ratio (AOR)	*p*	95% confidence interval for AOR	
			Lower	Upper
**Male gender**^**a**^	2.066	.003**	1.280	3.335
**BMI**	.920	.005**	.869	.975
**Life enhancement subscale score**	1.676	.012*	1.119	2.511
**Exercise milieu subscale score**	1.868	.017*	1.121	3.113
**Physical exertion subscale score**	1.580	.043*	1.014	2.461

^a^Reference female

*Significant *p* value < 0.05

**Significant *p* value < 0.01

The relation between benefits and barriers subscales scores and the occupation was evaluated where there was no significant difference between nurses and physicians as regards their benefit subscales scores with exception of psychological outlook subscale score where physicians showed higher score. Similarly, no significant difference was found between nurses and physicians as regard barrier subscales, except for the time expenditure score. As regards the tool overall score, physicians showed a higher score compared to nurses as shown in (Table 6).

**Table 6:** Comparison between nurses and physicians as regard benefits and barriers subscales scores

	Occupation		Test^#^	*p*
	Nurse, mean (SD)	Physician, mean (SD)		
**Benefits subscales**				
**Physical performance subscale score**	2.75 (0.51)	2.89 (.75)	1.871	.063
**Psychological outlook subscale score**	2.71 (0.55)	2.88 (.73)	2.147	.033*
**Preventative health subscale score**	2.71 (0.59)	2.80 (.64)	1.091	.276
**Life enhancement subscale score**	2.65 (0.46)	2.75 (.65)	1.45	.149
**Social interaction subscale score**	2.58 (0.55)	2.70 (.60)	1.532	.127
**Barriers subscales**				
**Physical exertion subscale score**	6.95 (1.45)	7.31 (1.81)	1.795	.075
**Time expenditure subscale score**	6.87 (1.8)	7.53 (1.89)	2.698	.007*
**Exercise milieu subscale score**	14.53 (2.79)	14.94 (3.08)	1.046	.296
**Family discouragement subscale score**	4.84 (1.25)	5.01 (1.27)	.996	.320
**Total benefits and barriers overall score**	111.28 (13.18)	116.44 (19.3)	2.656	.009*

^#^Independent *t* test

*Significant *p* value < 0.05

## Discussion

Among the 327 surveyed healthcare providers, the prevalence of physical activity was 44.6%. As regards the intention of exercise in the future, 28.8% of those physically inactive had the intention to start, while 26.6% were non-exercisers and they did not have the intention to start. Higher rates of physical activity were reported by different studies. For example, in Australia (70% of doctors and medical students) [21], in Saudi Arabia (65.2% of physicians) [22], in South India (64% of physicians) [23], in Northern Ireland (56.6% of general practitioner) [24], and in Ain Shams University, Egypt (75% of medical students) [25].

However, lower physical activity rates were reported in two studies in Saudi Arabia (21% of physicians Saudi Board residents in Aseer region, and 25.7% of family medicine program trainees in Eastern province); in the Faculty of Medicine at Ain Shams University, Egypt (16% of physicians); and in Mexico (42% of the physicians) were physically active [26–29].

Regarding factors associated with physical exercise engagement, our data revealed that the male gender was significantly associated with exercising. These results do not match with previous studies where males and females did not show a significant difference as regards physical activity [22, 25, 27, 30].

Overweight and obesity rate (48.7%) in our study was similar to that reported among medical students at Ain Shams University in the previous study; but lower than that reported among HCPs in Nigeria, as 72% were obese and overweight.

In our study, there was a significant difference between different BMI categories as regards practicing exercise where under- and normal-weight participants were engaged in physical exercise more than those who were overweight and obese. This is inconsistent with what was found in a study done on family medicine residents in Saudi Arabia where physical activity level did not vary according to the physicians' BMI [25, 27, 31].

Our data showed no difference between physicians and nurses as regards practicing exercise. This situation differs from that reported by other studies where physicians (62%) practiced exercise more than nurses (52%) [32].

Results of the current study revealed that among all participants, the top exercise benefits reported were decreased stress, increased muscle strength, improved body shape, and improved mental health. The significant benefits that affect exercise practicing were enjoyed exercise, decreased stress, improved mental health, felt relax, prevent heart attacks, live longer, physical endurance, good entertainment, improved disposition, self-concept, less tired, and improved work quality. However, these benefits do not match the earlier published results where the most commonly mentioned benefits by medical students were enhanced fitness, stamina, improved cardiovascular system, and decreased stress, while student nurses rated the highest benefits as improved fitness, improved cardiovascular system, muscle strength, and stamina [32].

On the other hand, the top exercise barriers reported were worn funny clothes, embarrassed to exercise, need time for family, and exercise is hard work. The significant barriers that affected exercise practicing were far exercise place, the cost of exercise, exercise is tiring, exercise is fatiguing, and exercise is hard work. Similar barriers were reported in previously conducted studies and it was similar among medicine and nursing students. Other barriers were reported in other studies as not having time to be physically active, the cost of participation, and feeling tired [23, 32–35].

Similar to previous studies findings, the present study shows that the exercising participants were reporting more benefits and fewer barriers to doing exercise. Also, nurses perceived less benefit and more barriers than physicians [32, 35].

### Study limitations

The use of a cross-sectional design limited the ability to assess the temporal relationship of physical exercise with different risk factors. Convenience sampling has also its drawbacks. Furthermore, our research was carried out only on participants in educational tertiary hospital related to the faculty of medicine located in the capital city; therefore, participants may be more conscious about exercise benefits and barriers. Hence, further studies should be performed involving varied healthcare providers in other types of healthcare facilities.

## Conclusion

The prevalence of regular exercise is low and inversely related to the female gender, BMI, physical exertion, and exercise milieu barriers subscale scores and directly related to life enhancement benefit subscale score.

### Recommendations

Health education programs about exercise benefits and barriers targeting healthcare workers should be done to improve their role modeling to patients through adopting a healthy lifestyle and improving counseling skills to patients. Physical exercise promotion initiatives might also be beneficial.

## Data Availability

The datasets generated during the current study are available on request

## Abbreviations

HCPs: Healthcare providers
BMI: Body mass index
PA: Physical activity
WHO: World Health Organization
SoC: The Stages of Change short-form questionnaire
EBBS: The Exercise Benefits and Barriers Scale
